# General vs. neuraxial anaesthesia in hip fracture patients: a systematic review and meta-analysis

**DOI:** 10.1186/s12871-017-0380-9

**Published:** 2017-06-28

**Authors:** Julia Van Waesberghe, Ana Stevanovic, Rolf Rossaint, Mark Coburn

**Affiliations:** Department of Anaesthesiology, University Hospital RWTH Aachen, Pauwelsstraße 30, 52074 Aachen, Germany

**Keywords:** Hip fracture, Neuraxial anaesthesia, General anaesthesia, 30-day mortality, In-hospital mortality, Length of hospital stay

## Abstract

**Background:**

Hip fracture is a trauma of the elderly. The worldwide number of patients in need of surgery after hip fracture will increase in the coming years. The 30-day mortality ranges between 4 and 14%. Patients’ outcome may be improved by anaesthesia technique (general vs. neuraxial anaesthesia). There is a dearth of evidence from randomised studies regarding to the optimal anaesthesia technique. However, several large non-randomised studies addressing this question have been published from the onset of 2010.

**Methods:**

To compare the 30-day mortality rate, in-hospital mortality rate and length of hospital stay after neuraxial (epidural/spinal) or general anaesthesia in hip fracture patients (≥ 18 years old) we prepared a systematic review and meta-analysis. A systematic search for appropriate retrospective observational and prospective randomised studies in Embase and PubMed databases was performed in the time-period from 01.01.2010 to 21.11.2016. Additionally a forward searching in google scholar, a level one reference list searching and a formal searching of trial registries was performed.

**Results:**

Twenty retrospective observational and three prospective randomised controlled studies were included. There was no difference in the 30-day mortality [OR 0.99; 95% CI (0.94 to 1.04), *p* = 0.60] between the general and the neuraxial anaesthesia group. The in-hospital mortality [OR 0.85; 95% CI (0.76 to 0.95), *p* = 0.004] and the length of hospital stay were significantly shorter in the neuraxial anaesthesia group [MD -0.26; 95% CI (−0.36 to −0.17); *p* < 0.00001].

**Conclusion:**

Neuraxial anaesthesia is associated with a reduced in-hospital mortality and length of hospitalisation. However, type of anaesthesia did not influence the 30-day mortality. In future there is a need for large randomised studies to examine the association between the type of anaesthesia, post-operative complications and mortality.

## Background

The worldwide number of hip fractures in elderly patients will rise due mainly to the demographic change from 1.66 million in the year 1990 to 6.25 million in the year 2050 [1]. Furthermore, elderly hip fracture patients present an array of comorbidities which are associated with an increased risk of morbidity and mortality [2]. The one-month mortality ranges from 4 to 14% [3–6].

Thus far, the ideal anaesthetic technique (general vs. neuraxial anaesthesia) has not been identified. The most recent randomised studies were condensed in a meta-analysis which was performed in the year 2016. The systematic review of Guy and colleagues included 31 randomised studies published between 1977 and 2013 [7]. However, only 28 studies comprising 2.976 patients could be included for the meta-analysis. Therefore, there is a high bias risk. Obviously the studies have been incapable of addressing, for example, a distinction in the 30-day mortality. Furthermore, there has been a change in clinical practice since 1977 [7]. However, since 2010 several large scale non-randomised studies have been published [8–27]. The objective of the present systematic review and meta-analysis is to provide a six-year overview of the literature assessing the influence of the anaesthetic technique for hip fracture surgery in prospective randomised and retrospective observational studies.

## Methods

### Protocol and registration

The study protocol has not been previously published. The manuscript has been prepared according to criteria of the PRISMA checklist and guidelines for systematic reviews and meta-analyses [28]. This systematic review and meta-analysis was registered in the international prospective register of systematic reviews (Prospero: CRD42016033254).

### Eligibility criteria

Before carrying out the systematic review and meta-analysis the exclusion and inclusion criteria were pre-defined by all authors. We included only human studies, which were published between 01.01.2010 and 21.11.2016 and assessed advantages of the applied anaesthetic technique general vs. neuraxial anaesthesia (epidural or spinal) in adult (≥ 18 years old) hip fracture patients. Prospective randomised and observational studies were included, which addressed the 30-day mortality, in-hospital mortality or length of hospital stay. As secondary outcome we examined the postoperative incidence of myocardial infarction, pneumonia, pulmonary embolism and respiratory failure after hip fracture surgery. We excluded case series and systematic reviews. Studies of all languages were included in the search.

### Information sources and search

In March 2017 a systematic search was performed via the database PubMed and Embase. The search term “anesthesia and hip fracture” or “anaesthesia and hip fracture” was used in both databases. Additionally, one study was included which was not identified via the systematic literature search [10]. The full search strategy for PubMed was: ((“anaesthesia”[All Fields] OR “anesthesia”[MeSH Terms] OR “anesthesia”[All Fields]) AND (“hip fractures” [MeSH Terms] OR (“hip” [All Fields] AND “fractures” [All Fields]) OR “hip fractures” [All Fields] OR (“hip” [All Fields] AND “fracture” [All Fields]) OR “hip fracture” [All Fields])) AND (“2010/01/01” [PDat]: “2016/11/21” [PDat]) and for Embase: ((AU = Anesthesia? OR (Anesthesia#)) AND (AU = HIP? OR ((HIP#)) AND (AU = FRACTURE? OR (FRACT####))) AND PY = 2010 to 2016. Additionally, a forward searching in google scholar, a level one reference list searching and a formal searching of trial registries (https://clinicaltrials.gov/; www.who.int/ictrp/en/ (international clinical trials registry platform) Search Portal of the World Health Organization) was performed. The results of the study of White and colleagues for the hospital length of stay was provided by one of the Co- authors [26].

### Study selection and data collection

JVW conducted the literature search and screened all hits based on the full text. Additionally, MC and AS verified all hits for eligibility independently. Only human studies, prospective randomised and retrospective observational studies were included. Case series and systematic reviews were excluded.

### Data items

A standardised table based on the PICO approach was made to reveal the salient results [28]. It contains the study type, applied anaesthetic technique, the sample size, primary and secondary outcome variables, summarized results and conclusion. We carried out a meta-analysis for the 30-day mortality, the in-hospital mortality and the length of hospital stay. In addition, we assessed as secondary outcome the postoperative incidence of myocardial infarction, pneumonia, pulmonary embolism and respiratory failure and performed a meta-analysis.

### Assessment of risk of bias

In order to assess bias risk, the Cochrane Collaboration’s tool for randomised studies was applied. The five domains of bias were classified as high, moderate or low risk. Regarding the non-randomised studies the Cochrane ACROBAT-NRSI tool was used. The seven domains of bias were also classified as high, moderate or low risk, accordingly.

### Statistics

The meta-analyses were performed using the RevMan 5.3 software. Due to the clinical and methodological heterogeneity of the included studies a random-effects model was applied for the meta-analysis. *P*-values less than 0.05 were regarded as statistically significant in the seven meta-analyses. The standard deviation (SD) was calculated, if not mentioned, based on the range (Maximum-Minimum)/4 or based on the 95% confidence interval [SD = √N× (upper limit-lower limit)/3.92)] or on the Interquartile Range (IQR) [SD = IQR/1.35], whichever was available.

## Results

### Study selection

**Fig. 1:**
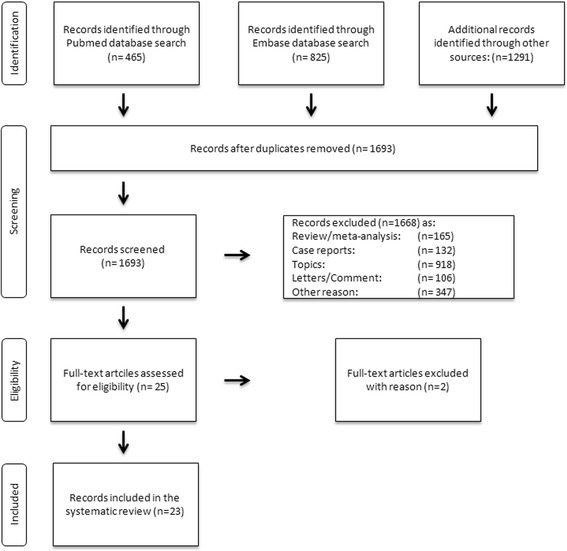
Prisma-flow diagram for the literature search and exclusion criteria

The search in PubMed identified 465 and in Embase 825 studies. The forward searching in google scholar revealed 538 studies, the list one reference searching 662 studies and the formal searching of trials registries 91 studies (clinicaltrials.gov *n* = 71; ICTRP *n* = 20) After removing the duplicates, we screened 1693 studies. Case reports, systematic reviews and meta-analyses were excluded. The study from Helwani and colleagues was included without being identified via our search term (“anesthesia and hip fracture” and “anaesthesia and hip fracture”) [10]. Finally 25 full text articles were assessed for eligibility. Two full-text articles were excluded as the outcome parameters did not fit the outcome variables of this study. One study did not define the meaning of the term “local anesthesia”, another study described the postoperative length of stay without usable values for our systematic review. The aim of Basques and colleagues was to identify factors, associated with an increased length of stay after hip fracture surgery, like the type of surgery [29, 30]. In total 23 studies were included for this systematic review and meta-analysis, see Fig. 1. [8–27, 31–33].

### Study characteristics/participants

Twenty retrospective observational studies and three randomised controlled studies were included [8–27, 31–33]. Overall 413.999 patients were analysed in this systematic review and meta-analysis. 249.408 patients received general anaesthesia and 150.964 patients received neuraxial anaesthesia (spinal anaesthesia and in some cases epidural anaesthesia). Our aim was to include only adult patients with a hip fracture over than 18 years. The study of Rashid and colleagues included patients with an age of 14–98 years. The mean age in the study was 65. Therefore, we decided to include this study in our systematic review [17]. Sample sizes in the included studies varied widely. The largest study included 104.088 patients and the smallest one 45 patients, see Table 1 [25, 32].

**Table 1:** Results of the 21 included studies

Author/Reference	Study type	Anaesthesia	Sample size	Primary outcome	Secondary outcome	Results	Conclusion
Basques et al. 2015 [8]	Retrospective observational study	GA vs. SA	*n* = 9.842 GA = 7.253 (73.7%) SA = 2.589 (26.3%)	Operating time; length of stay (LOS); adverse events within 30 post-operative days, rate of re-admission	30-day mortality	30- day mortality: GA vs. SA: (OR 0.98, 95% CI 0.82 to 1.20, *p* = 0.908); LOS: GA vs SA: (HR: 1.28, 95% CI 1.22 to 1.34, *p* < 0.001)	There was no difference between the groups except of length of hospital stay.
Biboulet et al. 2012 [32]	Randomised controlled study	GA vs. SA (propofol, sevoflurane)	*n* = 45 GA = 30 SA = 15	Blood pressure profile, heart rate profile	30-day mortality	Hypotension episodes: SA = 0 (range, 0-6) vs. propofol = 11.5 (range 1-25) vs. sevoflurane = 10 (range,1-23) (*p* < 0.0001); maximal decrease in arterial pressure: SA = 26% [16], propofol = 47% [8%], sevoflurane = 46% [12%] (*p* < 0.001)	SA in elderly patients provided better blood pressure stability than propofol and sevoflurane.
Brox et al. 2016 [23]	Retrospective observational study	GA vs. SA vs. mixed	*n* = 7.585 GA = 4.257 (56%) SA = 3.059 (40%) Mixed = 269 (4%)	30-day, 90-day and 365-day mortality		30-day mortality: GA = 177 (4%) vs. SA = 113 (4%) vs. mixed = 17 (6%); 90-day mortality: GA = 336 (8%) vs. SA = 224 (7%) vs. mixed = 23 (9%); 365-day mortality: GA = 661 (16%) vs. SA = 424 (14%) vs. mixed = 41 (15%)	There was no difference between the groups.
Chu et al. 2015 [25]	Retrospective observational study	GA vs. NA (spinal/epidural)	*n* = 104.088 GA = 52.044 NA = 52.044	In-hospital mortality	Acute stroke, transient ischemic stroke, acute myocardial infarction, acute respiratory failure, acute renal failure	In-hospital death: GA vs. NA: 1.363 (2.62%) vs. 1.107 (2.13%), *p* < 0.001	The GA group had a greater percentage and higher odds of adverse in-hospital events than the NA group.
Fields et al. 2010 [9]	Retrospective observational study	GA vs. SA	*n* = 6.133 GA = 4.318 (72.6%) SA = 1.815 (27.4%)	30-day complications, 30-day mortality	none	SA vs. GA: 30-day mortality: (6.67 vs. 5.84, *p* = 0.21); overall complications: (45.75% vs. 48.97%, *p* = 0.001)	GA had a higher risk of 30-day complications compared to SA. There was no difference related to mortality.
Heidari et al. 2011 [33]	Randomised controlled study	GA vs. NA (EA/SA)	*n* = 270 GA = 197 NA = 190	30-day mortality, in-hospital mortality, Length of hospital stay, postoperative complications	None	30-day mortality: GA vs. NA: 0 vs. 2, *p* = 0.299; In-hospital mortality: GA vs. NA: 5 vs. 0, *p* = 0.107; Length of hospital stay: GA vs. NA: 4.3 (1.8) vs. 3.8 (1.6), *p* = 0.042	The length of hospital stay was significantly longer in the GA group. The morbidity and mortality rates were similar in both groups.
Helwani et al. n [10]	Retrospective observational study	GA vs.NA (SA/EA)	*n* = 12.929 GA = 7.826 (60.5%) NA = 5.103 (39.5%) [SA = 4.377 (85.8%); EA = 126 (2.5%)]	30-day mortality, LOS, deep surgical site infection (dssi), cardiovascular (cvc) -, pulmonary complications (pc)	None	NA vs. GA: dssi: (OR = 0.38; 95% CI = 3% to 7%; *p* < 0.001); LOS: (OR = 0.73; 95% CI = 0.68 to 0.89, *p* < 0.001); 30-day mortality: (OR = 0.78; 95% CI = 0.43 to 1.42; *p* > 0.05); cvc: (OR = 0.61; 95% CI = 0.44 to 0.85; *p* < 0.001); pc: (OR = 0.51; 95% CI = 0.33 to 0.81; *p* < 0.001)	NA was associated with a reduction in dssi rates, LOS, rates of postoperative cvc and pc. There was no difference in the mortality between NA and GA.
Karademir et al. 2015 [11]	Retrospective observational study	GA vs. SA	*n* = 11 GA = 30 (26%) SA = 85 (74%)	1-year mortality rate	None	RA vs. GA: *p* = 0.63	No significant difference in the 1-year mortality between GA and SA group
Karaman et al. 2015 [12]	Retrospective observational study	GA vs. NA (SA/EA)	*n* = 308 GA = 105 (34.1) NA = 203 (65.9%)	Overall-mortality	None	mortality rate: GA (*n* = 40) vs. NA (*n* = 37) (*p* < 0.01); OR: 2.761 (95% CI:1.62 to 4.69)	The mortality rate of patients receiving GA was higher than mortality rate of patients receiving NA.
Kim et al. 2013 [13]	Retrospective observational study	GA vs. SA vs. EA	*n* = 506 GA = 246 (48.62%) SA = 249 (49.21%) EA = 11 (2.17%)	30-day mortality, pulmonary complications (pc); cardiac complications (cc); Delirium	None	30-day mortality: GA = 7 (2.8%); SA = 4 (1.6%); EA = 0 (0.0%); *p* = 0.57; pc: GA = 91 (37.1%), SA = 74 (29.8%), EA = 3 (27.3%), *p* = 0.20; cc: GA = 4 (1.6%), SA = 2 (0.8%), EA = 0 (0.0%) *p* = 0.52; Delirium: GA = 31 (12.7%), SA = 27 (10.9%), EA = 1 (9.1%), *p* = 0.8	Methods of anaesthesia did not influence mortality and postoperative complications.
Le-Wendling et al. 2012 [14]	Retrospective observational study	GA vs. RA (single injection spinal, continuous spinal, continuous epidural) with or without continuous nerve block	*n* = 308 GA = 235 (76.30%) RA = 73 (23.70%)	In-hospital mortality, hospitalization costs (hc), Length of stay (LOS)	Re-hospitalization	hc: RA vs. GA ($ 16.789 + 631 vs. $ 16.815 + 643, *p* = 0.9557); LOS: 6.4 vs. 6.6 days, *p* = 0.64; in-hospital mortality: 2 (2.74) vs. 9 (3.83)	There was no difference in postoperative morbidity, rates of re-hospitalization, in-patient mortality or hc in patients receiving RA or GA.
Neuman et al. 2012 [27]	Retrospective observational study	GA vs. NA	*n* = 18.158 GA = 12.904 NA = 5.254	In-hospital mortality	Pulmonary and cardiovascular complications	In-hospital mortality: GA vs. NA: 325 (2.5%) vs. 110 (2.1%), *p* = 0.090	The mortality rate was similar between the two groups.
Neuman et al. 2014 [15]	Retrospective observational study	GA vs. NA (spinal/epidural)	*n* = 56.729 GA = 40.825 (72%) NA = 15.904 (28%)	30-day mortality	Length of stay (LOS)	30-day mortality: NA = 5.3%, GA = 5.4% (difference 0.1%; 95 CI −0.5 to 0.3; *p* = 0.55); LOS: RA = 6 days (95% CI: 6 to 6.1) vs. GA = 6.3 days (95% CI: 6.2 vs. 6.3), difference LOS: −0.2 days (95% CI: −0.3 `to 0.2; *p* < 0.001)	30-day mortality did not differ significantly between GA and NA. NA was associated with a shorter LOS.
Parker et al. 2015 [31]	Randomised controlled study	GA vs. SA	*n* = 322 GA = 164 (50.93%) SA = 158 (49.07%)	Mortality after 30, 90, 120 and 365 days	Surgical outcome, general complications, hospital stay (LOS)	30-day mortality: GA vs SA (4.9% vs. 3.2%; *p* = 0.57); 90 days: (7.3% vs 7.6%; *p* = 1.00); 120 days: (7.3% vs 7.6%; *p* = 0.55); 365 days: (11.7% vs 20.2%; *p* = 0.05); LOS in days (standard deviation): GA = 15.9 (13.7); RA = 16.2 (14.6); *p* = 0.75	No differences between GA and SA.
Patorno et al. 2014 [16]	Retrospective observational study	GA vs NA (spinal/epidural) vs. GA + NA	*n* = 73.284 GA = 61.554 (84.0%) NA = 6.939 (9.47%) GA + NA = 4.791 (6.53%)	In-hospital mortality	none	In-hospital mortality: GA vs. NA: (risk ratio 0.93, 95% CI 0.78 to 1.11)	Mortality risk did not differ significantly between GA and NA.
Rashid et al. 2013 [17]	Retrospective observational study	GA vs. NA (epidural/spinal)	*n* = 194 GA = 107 (55.15%) NA = 87 (44.85%)	Operating time, length of stay (LOS), blood loss, mortality	none	Operative time: GA = 1.54 ± 0.6, NA = 1.24 ± 0.39, *p* < 0.01; LOS: GA = 9.35 ± 9.0, NA = 8.63 ± 3.6, *p* = 0.484; blood loss: GA = 928 ± 360, NA = 912 ± 400, *p* = 0.758; mortality: GA = 4, NA = 5	There were no differences between LOS, blood loss and mortality. The only significant difference was in the operating time.
Shih et al. 2010 [18]	Retrospective observational study	GA vs. SA	*n* = 335 GA = 167 (49.85%) SA = 168 (50.15%)	perioperative morbidity, duration of surgery, length of stay (LOS), blood loss	none	GA vs. SA: duration: 165 min. vs. 150 min.; *p* < 0.001, LOS: 9 days vs. 8 days, *p* = 0.04); overall mortality: (5/167 [3%] vs. 2/168 [1.2%]; *p* = 0.25); overall morbidity: (21/167 [12.6] vs. 9/168 [5.4%]; *p* = 0.02)	GA increased the risk of postoperative morbidity in octogenarian patients after hip fracture repair. Patients with pre-existing respiratory diseases were especially vulnerable.
Seitz et al. 2014 [19]	Retrospective observational study	GA (inhalational, intravenous, GA combined with epidural or local anaesthesia) vs. SA	*n* = 20.973 GA = 8.818 (42.1%) SA = 12.155 (57.9%)	30-day mortality, 30-day postoperative medical complication, ICU till 7 days after surgery, length of stay (LOS)	none	GA vs. SA: 30-day mortality: GA = 691 (11.3%) vs. SA = 665 (10.8%), *p* = 0.44; ICU: GA = 371 (6%) vs.SA = 259 (4.2%), *P* < 0.001, 30-day postoperative medical complication: GA = 1.165 (19%), SA = 1.169 (19%) *p* = 0.92; LOS in days (± standard deviation): GA = 16.1 (20.2), SA = 16.0 (23.6), *p* = 0.72	GA and SA were associated with similar rates of most postoperative events.
Sevtap et al. 2013 [20]	Retrospective observational study	GA vs. SA vs. EA	*n* = 185 GA = 67 (36.21%) SA = 67 (36.21%) EA = 51 (27.58%)	7-day mortality, 30-day-mortality	Blood loss, blood transfusion, length of stay (LOS)	7-day mortality: GA = 3 (4.4%), SA = 2 (2.9%), EA = 1 (1.9%), *p* = 0.738; 30-day mortality: GA = 4 (1.4%), SA = 6 (5.9%), EA = 4 (5.8%), *p* = 0.805; LOS: GA = 13.6 ± 8.9, SA = 12.5 ± 5.2, EA = 15.7 ± 9.4, *p* = 0.228	There was no difference in the 7-day and 30-day mortality between the anaesthesia techniques. Further there were no differences in the other factors.
Tung et al. 2016 [24]	Retrospective observational study	GA vs. RA (epidural/spinal)	*n* = 17.189 GA = 6.063 (35.1%) NA = 11.153 (64.9%)	30-day all-cause mortality, 30-day all cause readmission, 30-day specific cause readmission		30-day mortality: GA = 104 (1.7%), NA = 189 (1.7%), *p* = 0.891, (OR 0.89, 95% CI [0.67 to 1.18], *p* = 0.409) 30-day readmission all-cause: GA = 771 (12.8%), NA = 1332 (12%), (OR 0.83, 95% CI 0.75 to0.93, *p* = 0.001), Surgical site infection readmission: (OR 0.69, 95% CI 0.49 to 0.97, *p* = 0.031)	There was no difference in the 30-day mortality between the two groups. NA is associated with a decreased 30-day all-cause readmission and surgical site infection readmission compared to GA
White et al. 2014 [21]	Retrospective observational study	GA vs. SA	*n* = 65.535 GA = 35.373 (53.97%) SA = 23.665 (36.11%)	30-day mortality	none	30-day mortality: GA = 1.066 (7.0%) vs. SA = 1.345 (7.3%); *p* = 0.053	No differences between GA and SA.
White et al. 2016 [26]	Retrospective observational study	GA vs. SA (with or without peripheral nerve block)	*n* = 11.085 GA = 985 GA with block = 4.364 GA + SA = 458 SA = 1.506 SA with block = 3.234 Unknown = 538	30-day mortality Length of stay (IQR [range]) Intraoperative blood pressure	None	30-day mortality: GA vs. SA: 53 (5.4) vs. 87 (5.8) LOS: GA (*n* = 883): 13.2 (8.0-23.4 [1.3-165.8]) vs. SA (*n* = 1.319): 13.2 (8.0-22.8 [0.2-287.9])	There was no significant difference in the 30-day mortality and the length of stay between the groups.
Whiting et al. 2015 [22]	Retrospective observational study	GA vs. NA	*n* = 7.764 GA = 5.840 (75.2%) NA = 1.924 (24.8%) (SA = 1.813 (23.4%); nerve block = 111 (1.4%))	Minor complications, major complications, total complications within 30-day postoperative; 30-day mortality	none	SA vs GA: minor complications (OR: 1.43; CI 95%: 1.15 to 1.77; *p* = 0.001), major complications (OR: 1.01; CI 95%: 0.81 to 1.24; *p* = 0.950), total complications (OR: 1.24; CI 95%: 1.05 to 1.48; *p* = 0.014), 30-day mortality (OR: 1.20; CI 95%: 0.92 to 1.56; *p* = 0.169)	NA was associated with significantly greater odds of minor and total perioperative complications compared with GA.

*cvc* cardiovascular complications, *dssi* deep surgical site infection, *EA* epidural anaesthesia, *GA* general anaesthesia, *hc* hospitalization costs, *LOS* length of hospital stay, *NA* neuraxial anaesthesia, *pc* pulmonary complications, *RA* regional anaesthesia, *SA* spinal anaesthesia

### Risk of bias within and across studies

Analyses of the risk of bias for retrospective observational studies and randomised controlled studies are described in Tables 2 and 3, respectively.

**Table 2:** Risk of bias of the retrospective studies

Author	Bias due to confounding	Bias in selection of participants	Bias in measurement of intervention	Bias due to departures from intended interventions	Bias to missing data	Bias in measurement of outcome	Bias in selection of the reported result	Overall bias
Basques et al. 2015 [8]	Low risk	High risk	High risk	Low risk	Low risk	Low risk	Low risk	Moderate risk.
Explanation	The authors used calculated propensity scores to mitigate the selection bias.	Comparison group was retrospectively determined according to anaesthesia technique (SA vs. GA).	The ACS-NSQIP database does not capture the type or anaesthetic dosage used.	It is a retrospective study. There were no departures from intended interventions.	No important data missing.	The database was filled with data from medical records and interviews by trained reviewers.	The authors used bivariate and propensity-adjusted multivariate regression analyses. Binary outcomes were compared using logistic regression.	
Brox et al. 2016 [23]	Low risk	High risk	High risk	Low risk.	Low risk	High risk	Low risk.	High risk.
Explanation	The authors used Pearson’s chi-squared test and the Kruskal-Wallis test to mitigate selection bias.	Comparison group was retrospectively determined according to anaesthesia technique (SA vs. GA).	The database does not capture the type or dose of anaesthetic used.	It is a retrospective study. There were no departures from intended interventions	No important data missing.	A hip fracture registry was used to identify the patients. No information about the people collecting the data.	The authors used a multivariable conditional logistic regression model.	
Chu et al. 2015 [25]	Low risk	High risk	High risk	Low risk.	Low risk	Unclear risk	Low risk	Moderate risk
Explanation	The author used calculated propensity score to mitigate the selection bias.	Comparison group was retrospectively determined according to anaesthesia technique (GA vs. NA).	The database does not capture the type or dose of anaesthetic used.	It is a retrospective study. There were no departures from intended interventions	No important data missing.	A database was used without information of the people	The author used a propensity score, Student t test, Pearson chi-square test.	
Fields et al. 2010 [9]	Low risk	High risk	High risk	Low risk	Low risk	Low risk	Low risk	Moderate risk
Explanation	The authors used calculated propensity scores to mitigate the selection bias.	Comparison group was retrospectively determined according to anaesthesia technique (SA vs. GA).	The database does not capture the type or dose of anaesthetic used.	It is a retrospective study. There were no departures from intended interventions.	No important data missing.	A surgical clinical reviewer at each hospital collects the data.	The authors used a multivariate logistic regression.	
Helwani et al. 2015 [10]	Low risk	High risk	High risk	Low risk	Low risk	Low risk	Low risk	Moderate risk
Explanation	The authors used a propensity score to reduce the selection bias	Comparison group was retrospectively determined according to anaesthesia technique (GA vs. NA).	The database does not capture the type or dose of anaesthetic used.	It is a retrospective study. There were no departures from intended interventions.	No important data missing.	Dedicated data personnel collect, validate and submit the data after rigorous uniform training and examination.	Demographic and clinical characteristics were compared between the two groups by using Pearson chi-square test for all categorical variables.	
Karademir et al. 2014 [11]	High risk	High risk	High risk	Low risk	High risk	High risk	Low risk	High risk
Explanation	Retrospective study with high risk of confounders	Comparison group was retrospectively determined according to anaesthesia technique and the surgery technique (GA vs. NA).	The database does not capture the type or dose of anaesthetic used.	No departure from intervention.	No exact data about the mortality rate in the NA and the GA group.	The data were recruited from hospital data base and patient files.	The authors used a survival analysis by Kaplan-Meier method and a cox regression model.	
Karaman et al. 2015 [12]	High risk	High risk	High risk	Low risk	Low risk	High risk	Low risk	High risk
Explanation	Retrospective study with high risk of confounders.	Comparison group was retrospectively determined according to anaesthesia technique (GA vs. NA anaesthesia).	The dose and type of anaesthetic used is not described.	It is a retrospective study. There were no departures from intended interventions.	No missing data.	Patient screening was performed retrospectively from hospital electronic medical record system.	The authors used the Student t-test and the Yates Continuity Correction test to compare the results between the two groups.	
Kim et al. 2013 [13]	High risk	High risk	High risk	Low risk	Low risk	High risk	Low risk	High risk
Explanation	Retrospective study with high risk of confounders.	The three groups (GA vs. SA vs. EA) were retrospectively determined according to anaesthesia technique.	The study does not describe the dose and type of anaesthetic used. Especially the dose could differ between the individuals.	No departure from intervention.	No missing data.	No information about the way the results were collected.	The authors used a chi-square test, Fisher’s exact test and binary logistic regression analysis to review the results.	
Le-Wendling et al. 2012 [14]	Low risk	High risk	High risk	Low risk	Low risk	Low risk	Low risk	Moderate risk
Explanation	The authors used calculated propensity scores to mitigate the selection bias	The two groups (GA vs. RA) were retrospectively determined according to anaesthesia technique.	The study does not describe the dose and type of anaesthetic used. Especially the dose could differ between the individuals.	No departure from intervention.	No missing data.	The hospital service support analyst collected the data.	The authors used a multiple logistic regression model and a linear regression model to compare the result.	
Neuman et al. 2012 [27]	Low risk	High risk	High risk	Low risk	Low risk	Low risk	Low risk	Low risk
Explanation	The author used calculated propensity scores to mitigate selection bias.	The two groups (GA vs. NA) were retrospectively determined according to anaesthesia technique.	The study does not describe the dose and type of anaesthetic used. Especially the dose could differ between the patients.	No departure from intervention.	No missing data.	The results were collected in the New York State Inpatient Database which was overseen by the U.S. Agency for Healthcare.	The author used the Wilcoxon rank sum test and the chi-square test to compare the results.	
Neumann et al. 2014 [15]	Low risk	High risk	High risk	Low risk	Low risk	High risk	Low risk	High risk.
Explanation	The authors used near-far matching, standardized differences, across-hospitalmatch and a within-hospital match to reduce the selection bias.	The two groups (GA vs. EA) were retrospectively determined according to anaesthesia technique.	The study does not describe the dose and type of anaesthetic used.	No departure from intervention.	No missing data.	No information about the way the results were collected	The authors used an instrumental variable method, the McNemar test and the x^2^ statistic to compare the results.	
Patorno et al. 2014 [16]	High risk	High risk	High risk	Low risk	Low risk	Low risk	Low risk	High risk.
Explanation	Retrospective study with high risk of confounders.	The three groups (GA vs. NA, GA + NA) were retrospectively determined according to the anaesthesia technique.	The data does not capture the dosage and type of anaesthetic used. The does could differ between the patients.	No departure from intervention.	No missing data.	The authors used the Premier research database. The data were collected from member hospitals through Premier’s informatics products.	The authors used a multi-variable logistic regression to compare the results.	
Rashid et al. 2013 [17]	High risk	High risk	High risk	Low risk	Low risk	High risk	High risk	High risk
Explanation	Retrospective study with high risk of confounders.	The two groups (GA vs. NA) were retrospectively determined according to anaesthesia technique.	The study does not describe the dose and type of anaesthetic used. The does could differ between the patients.	No departure from intervention.	No missing data.	Unclear how the data were collected and how the clinical measurement was done.	The authors use SPSS version 19 for statistical analyses. However no information is given on the type of analysis	
Seitz et al. 2014 [19]	Low risk	High risk	High risk	Low risk	Low risk	Low risk	Low risk	Moderate risk.
Explanation	The authors used calculated propensity scores to mitigate the selection bias.	The two groups (GA vs. NA) were retrospectively determined according to anaesthesia technique.	The study does not describe the dose and type of anaesthetic used. The does could differ between the patients.	No departure from intervention.	No missing data.	The used data sets were linked using unique, encoded identifiers and analysed at the Institute for Clinical Evaluative Sciences (ICES).	The authors used the Wilcoxon rank-sum test and chi-square test to compare the results.	
Shih et al. 2010 [18]	High risk	High risk	Low risk	Low risk	Low risk	High risk	Low risk	High risk.
Explanation	Retrospective study with high risk of confounders.	The two groups (GA vs. NA) were retrospectively determined according to anaesthesia technique.	The measurement of intervention is well-defined.	No departure from intervention.	No missing data.	Unclear how the data were collected and how the clinical measurement was done.	The authors used Student t-test, X2 or Fisher exact test and logistic regression to compare the results.	
Sevtap et al. 2013 [20]	High risk	High risk	Low risk	Low risk	Low risk	High risk	Low risk	High risk.
Explanation	Retrospective study with high risk of confounders.	The three groups (GA vs. SA vs. EA) were retrospectively determined according to the anaesthesia technique.	The measurement of intervention is well-defined.	No departure from intervention.	No missing data.	This is a retrospective study. And all the data were obtained from the medical data.	The authors used the one-way analysis of variance test for normally distributed data and the Kruskal Wallis test for abnormally distributed data. The categorical variables were compared using the chi-square tests.	
Tung et al. 2016 [24]	Low risk.	High risk.	High risk.	Low risk.	Low risk.	High risk.	Low risk.	High risk.
Explanation	The authors used calculated propensity scores to mitigate the selection bias.	The two groups (GA vs. NA) were retrospectively determined according to anaesthesia technique.	The study does not describe the dose and type of anaesthetic used. The dose could be different between the patients.	No departure from intervention.	No missing data.	Data were collected in the National Health Insurance research database. No information about the persons collecting the information.	The authors used a generalized estimation equation logistic regression model and propensity score.	
White et al. 2014 [21]	High risk	High risk	High risk	Low risk	Low risk	Low risk	Low risk	High risk.
Explanation	Retrospective study with high risk of confounders.	The two groups (GA vs. SA) were retrospectively determined according to anaesthesia technique.	The study does not describe the dose and type of anaesthetic used.	No departure from intervention.	No missing data.	Data were collected by specially trained personnel employed by each eligible hospital.	The authors used a two-tailed chi-squared test without Yate’s correction and multivariable regression analysis.	
White et al. 2016 [26]	High risk.	High risk	High risk	Low risk	High risk	Low risk	Low risk	High risk
Explanation	Retrospective study with high risk of confounders.	The two groups (GA vs. SA) were retrospectively determined according to anaesthesia technique.	The study described the different volumes used for intrathecal injections. However the dose and type for the general anaesthesia or the peripheral nerve block were not described.	No departure from intervention.	16.904 patient records. However only 11.085 could be analysed.	Data were collected by specially trained personnel employed by each eligible hospital.	The authors used Fisher’s exact test, chi-squared, Wilcoxon and Haenzel tests.	
Whiting et al. 2015 [22]	High risk	High risk	High risk	Low risk	Low risk	Low risk	Low risk	High risk.
Explanation	Retrospective study with high risk of confounders	The two groups (GA vs. NA) were retrospectively determined according to anaesthesia technique.	The study does not describe the dose and type of anaesthetic used. The dose could be different between the patients.	No departure from intervention.	No missing data.	Data were collected at each hospital directly from patients medical records through risk-assessment nurses trained as Surgical Clinical Reviewers (SCR)	The authors used chi-square, Fischer’s exact test and multivariate models.	

*EA* epidural anaesthesia, *GA* general anaesthesia, *NA* neuraxial group, *RA* regional anaesthesia, *SA* spinal anaesthesia

**Table 3:** Risk of bias of the randomised studies

Author	Random sequence generation (selection bias)	Allocation concealment (selection bias)	Blinding of participants and personnel (performance bias)	Blinding of outcome assessment (detection bias)	Incomplete outcome data addressed (attrition bias)	Selective reporting (reporting bias)	Other bias
Biboulet et al. 2012 [32]	Unclear risk	Unclear risk	Unclear risk	Unclear risk	Low risk	Low risk	Unclear risk
Explanation	No information about the sequence generation process.	Method of concealment is not described.	Insufficient information about blinding of participants or personnel.	Insufficient information about blinding of outcome assessment.	No incomplete outcome data.	The paper included all expected outcome.	The authors described several limitations which could influence the outcome.
Heidari et al. 2011 [33]	Low risk	Unclear Risk	Low risk	Unclear risk	Low risk	Low risk	Unclear risk
Explanation	A random-number table was used.	Method of concealment is not described.	It was not possible to blind the patient or the anaesthetist.	Insufficient information about blinding of outcome assessment.	No incomplete outcome data.	The paper included all expected outcomes.	The author described several limitations, which could influence the outcomes.
Parker et al. 2015 [31]	Unclear risk	Unclear risk	High risk	Low risk	Low risk	Low risk	Unknown risk
Explanation	Randomisation was undertaken by the opening of sealed opaque numbered envelopes. The envelopes were prepared at the start of the study by a person independent to the study.	Randomisation was undertaken by the opening of sealed opaque numbered envelopes. The envelopes were prepared at the start of the study by a person independent to the study.	The exact technique and doses of the anaesthetic used was the choice of the anaesthetist. On the verge of surgery the patient knows if he gets a general or spinal anaesthesia.	There was no blinding of investigator, participants or outcome assessors without having influence on outcomes like 30-day mortality.	Attrition <1%.	No important outcomes missing.	Small numbers of patients being included.

### Results of individual studies

#### Mortality

**Fig. 2:**
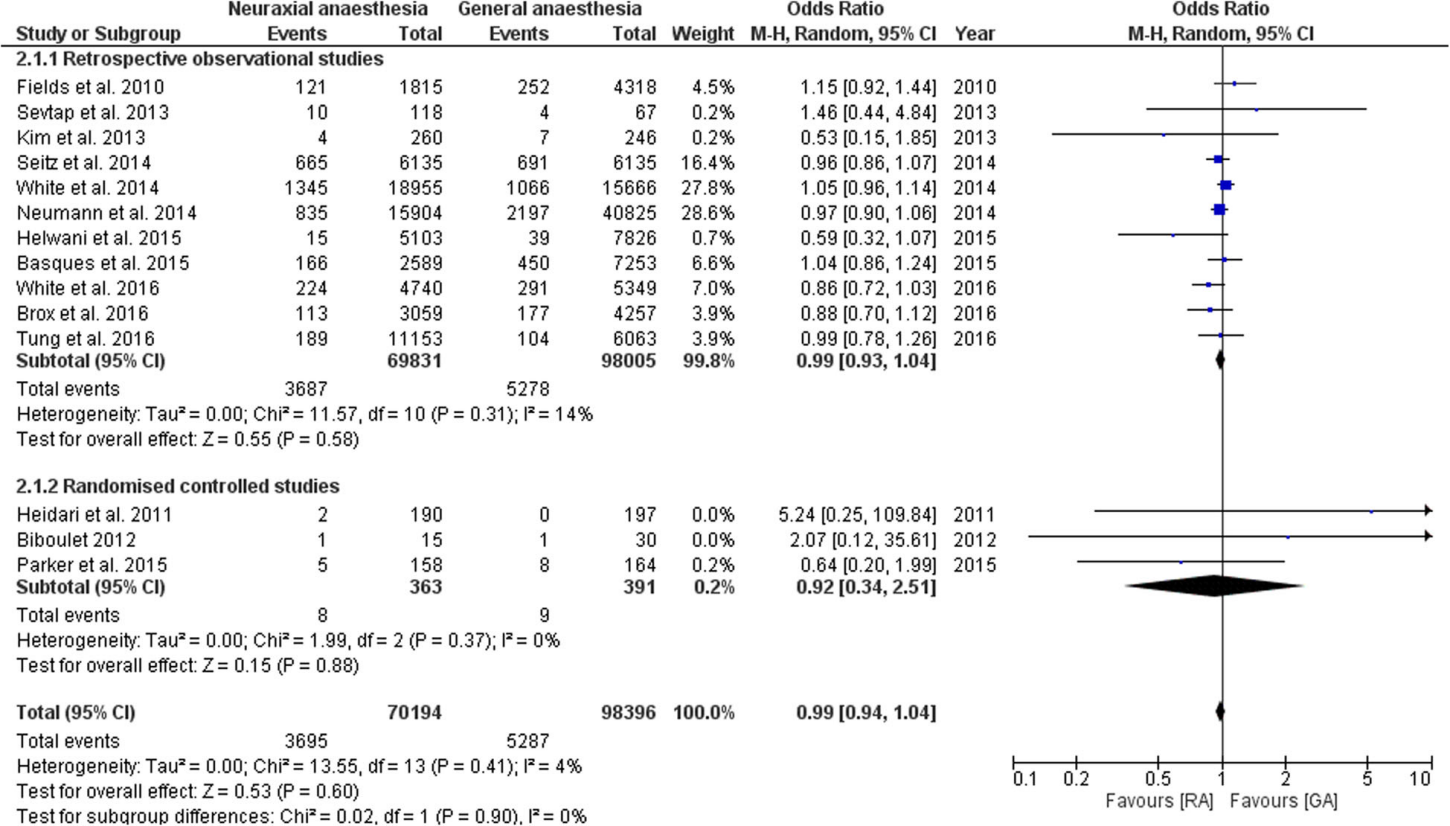
Meta-analysis of the 30 day mortality for the neuraxial anaesthesia group versus the general anaesthesia group calculated with random effect method. 2.1.1: Odds Ratio for the retrospective observational studies represented as subgroup. 2.1.2: Odds ratio for the randomised controlled studies represented as subgroup

Fourteen studies examined the effect of general vs. neuraxial anaesthesia on the 30-day mortality after hip fracture surgery in adults. Eleven were retrospective observational and three were prospective randomised studies [8–10, 13, 15, 19–21, 23, 24, 26, 31–33].All assessed studies could not identify a difference between the 30-day mortality, see Table 4. The meta-analysis including the fourteen studies revealed no significant difference for the 30-day mortality [Odds Ratio (OR) 0.99; 95% Confidence Interval (CI) (0.94 to 1.04), *p* = 0.60] [8–10, 13, 15, 19–21, 23, 24, 26, 31–33]. We performed a separate subgroup analysis of the eleven retrospective observational and the three randomised controlled studies. Both subgroup-analyses revealed no significant difference in the 30-day mortality between the two groups [OR 0.99, 95% CI (0.93 to 1.04), *p* = 0.58] vs. [OR 0.92, 95% CI (0.34 to 2.51), *p* = 0.88], see Fig. 2.

**Table 4:** Results of the 30-day mortality

Author/Reference	Study type	Anaesthesia	Sample size	Outcome parameter	Results	Conclusion
Basques et al. 2015 [8]	Retrospective observational study	GA vs. SA	*n* = 9.842 GA = 7.253 (73.7%) SA = 2.589 (26.3%)	30-day mortality	GA vs. SA: 6.2% vs. 6.4%; (OR 0.98, 95% CI 0.82 to 1.20, *p* = 0.908)	There was no difference related to the mortality.
Biboulet et al. 2012 [32]	Randomised controlled study	SA vs. GA (propofol, sevoflurane)	*n* = 45 GA = 30 SA = 15	30-day mortality	SA vs.GA: 1 (6.7%) vs. 1(7.1%), *p* = 0.76	There was no difference related to the mortality
Brox et al. 2016 [23]	Retrospective observational study	GA vs. SA	*n* = 7.585 GA = 4.257 (56%) SA = 3.059 (40%) Mixed = 269 (4%)	30-day mortality	30-day mortality: GA = 177 (4%) vs. SA = 113 (4%)	There was no difference related to the mortality.
Fields et al. 2010 [9]	Retrospective observational study	GA vs. SA	*n* = 6.133 GA = 4.318 (72.6%) SA = 1.815 (27.4%)	30-day mortality	SA vs. GA: 30-day mortality: 6.67% vs. 5.84%, *p* = 0.21	There was no difference related to the mortality.
Heidari et al. 2011 [33]	Randomised controlled study	GA vs. NA (Epidural/spinal)	*n* = 387 GA = 197 NA = 190	30-day mortality	GA vs. SA: 0 vs. 2, *p* = 0.299	There was no differencerelated to the mortality.
Helwani et al. 2015 [10]	Retrospective observational study	GA vs. NA(SA/EA)	*n* = 12.929 GA = 7.826 (60.5%) RA = 5.103 (39.5%) [SA = 4.377 (85.8%); EA = 126 (2.5%)]	30-day mortality	NA vs. GA: OR 0.78, 95% CI 0.43 to 1.42; *p* > 0,05	There was no difference in the mortality.
Kim et al. 2013 [13]	Retrospective observational study	GA vs. SA vs. EA	*n* = 506 GA = 246 (48.62%) SA = 249 (49.21%) EA = 11 (2.17%)	30-day mortality	GA = 7 (2.8%); SA = 4 (1.6%); EA = 0 (0.0%); *p* = 0.57	There was no difference in the mortality.
Neuman et al. 2014 [15]	Retrospective observational study	GA vs. NA (spinal/epidural)	*n* = 56.729 GA = 40.825 (72%) RA = 15.904 (28%)	30-day mortality	RA = 5.3%, GA = 5.4% (difference 0.1%; 95% CI 0.5 to 0.3; *p* = 0.55)	30-day mortality did not differ significantly between GA and NA.
Parker et al. 2015 [31]	Randomised controlled study	GA vs. SA	*n* = 322 GA = 164 (50.93%) SA = 158 (49.07%)	30-day mortality	30-day mortality: GA vs SA: 8 (4.9%) vs. 5 (3.2%); *p* = 0.57	There was no difference in the mortality.
Seitz et al. 2014 [19]	Retrospective observational study	GA (inhalational, intravenous, GA combined with epidural or local anaesthesia) vs. SA	*n* = 20.973 GA = 8.818 (42.1%) SA = 12.155 (57.9%)	30-day mortality	GA vs. SA: 30-day mortality: GA = 691 (11.3%) vs. SA = 665 (10.8%), *p* = 0.44	There was no difference in the mortality.
Sevtap et al. 2012 [20]	Retrospective observational study	GA vs. SA vs. EA	*n* = 185 GA = 67 (36.21%) SA = 67 (36.21%) EA = 51 (27.58%)	30-day-mortality	30-day mortality: GA = 4 (1.4%), SA = 6 (5.9%), EA = 4 (5.8%), *p* = 0.805	There was no difference in the mortality.
Tung et al. 2016 [24]	Retrospective observational study	GA vs. NA	*n* = 17.189 GA = 6.063 (35.1%) RA = 11.153 (64.9%)	30-day mortality	30-day mortality: GA = 104 (1.7%), NA = 189 (1.7%), *p* = 0.891, (OR 0.89, 95% CI 0.67 to 1.18, *p* = 0.409)	There was no difference in the mortality.
White et al. 2014 [21]	Retrospective observational study	GA vs. SA	*n* = 65.535 GA = 35.373 (53.97%) SA = 23.665 (36.11%)	30-day mortality	30-day mortality: GA = 1.066 (7.0%) vs. SA = 1.345 (7.3%); *p* = 0.053	No notable differences between GA and SA.
White et al. 2016 [26]	Retrospective observational study	GA vs. SA (with or without peripheral nerve block)	*n* = 11.085 GA = 985 GA with block = 4.364 GA + SA = 458 SA = 1.506 SA with block = 3.234 Unknown = 538	30-day mortality	30-day mortality: GA vs. SA: 291 (5.4%) vs. 224 (4.7%)	No notable differences between GA and SA.

*EA* epidural anaesthesia, *GA* general anaesthesia, *NA* neuraxial anaesthesia, *SA* spinal anaesthesia

Two studies assessed the overall mortality [12, 18]. Karaman and colleagues (*n* = 308, general anaesthesia = 105, neuraxial anaesthesia = 203) revealed that there is a higher mortality rate after receiving general anaesthesia than neuraxial anaesthesia [12]. Shih (*n* = 335, general anaesthesia = 167, neuraxial anaesthesia = 168) and colleagues indicated that the overall-mortality is not significantly different between the general anaesthesia and the neuraxial anaesthesia group. Of note, general anaesthesia increased the risk of overall-morbidity in patients after hip fracture surgery in this study. Patients with pre-existing respiratory diseases were especially vulnerable [18].

The retrospective study of Karademir and colleagues and the randomised controlled study of Parker and Griffiths examined the 1-year mortality. In both studies there is no significant difference in the 1-year mortality between the neuraxial and the general anaesthesia group [21, 31].

**Fig. 3:**
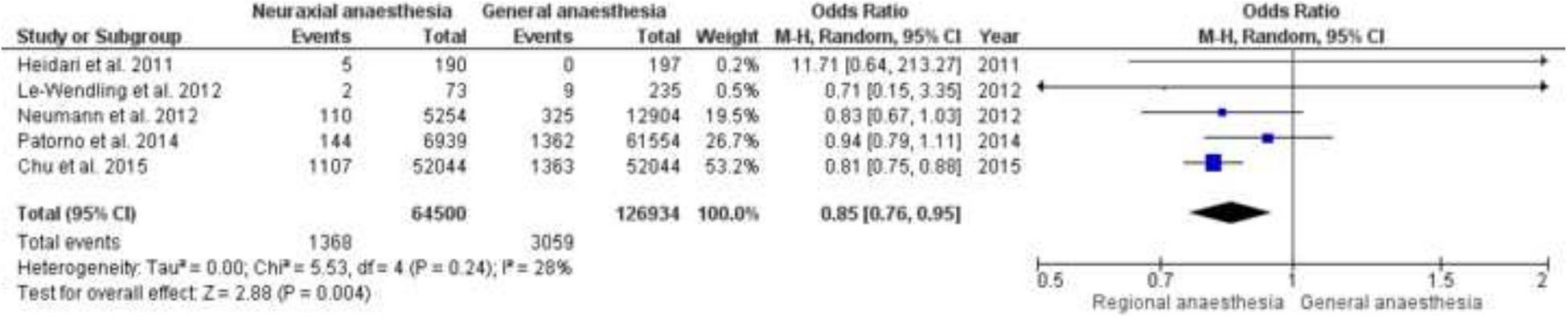
Meta-analysis of the in-hospital mortality for the neuraxial anaesthesia groups versus the general anaesthesia group. The odds ratio was calculated with a random effect method

The in-hospital mortality was examined by five studies, see Table 5. Four studies came to the result that the in-hospital mortality rate did not differ significantly between general and neuraxial anaesthesia [14, 16, 27, 33]. The study of Chu and colleagues (*n* = 104.088, general anaesthesia = 52.044, neuraxial anaesthesia = 52.044) revealed a significant higher incidence of the in-hospital mortality in the general anaesthesia group. Our meta-analysis, including the aforementioned five studies, showed a significant lower incidence of the in-hospital mortality in the neuraxial anaesthesia group. [OR 0.85; 95% CI (0.76 to 0.95), *p* = 0.004] with a negligible heterogeneity (I^2^ = 28%), see Fig. 3. [14, 16, 25, 27, 33].

**Table 5:** Results of the in-hospital mortality

Author/Reference	Study type	Anaesthesia	Sample size	Outcome parameter	Results	Conclusion
Chu et al. 2015 [25]	Retrospective observational study	GA vs. NA (spinal/epidural)	*n* = 104.088 GA = 52.044 NA = 52.044	In-hospital mortality	GA vs. NA: 1.363 (2.62%) vs. 1.107 (2.13%), *p* < 0.001	The incidence of on-hospital mortality was significantly lower in the NA group.
Heidari et al. 2011 [33]	Randomised controlled study	GA vs. NA (epidural/spinal)	*n* = 270 GA = 197 NA = 190	In-hospital mortality	GA vs. NA: 0 vs. 5, *p* = 0.107	The incidence of in-hospital mortality was similar in both groups.
Le-Wendling et al. 2012 [14]	Retrospective observational study	GA vs. RA (single injection spinal, continuous spinal, continuous epidural) with or without continuous nerve block	*n* = 308 GA = 235 (76.30%); RA = 73 (23.70%)	In-hospital mortality	RA vs. GA: 2 (2.74) vs. 9 (3.83)	There was no difference between the in-hospital mortality.
Neuman et al. 2012 [27]	Retrospective observational study	GA vs. NA	*n* = 18.158 GA = 12.904 NA = 5.254	In-hospital mortality	GA vs NA 325 (2.5%) vs. 110 (2.1%), *p* = 0.090	There was no difference for the in-hospital mortality between the two groups.
Patorno et al. 2014 [16]	Retrospective observational study	GA vs NA (spinal/epidural) vs. GA + NA	*n* = 73.284 GA = 61.554 (84.0%) NA = 6.939 (9.47%); GA + RA = 4.791 (6.53%)	In-hospital mortality	In-hospital mortality: GA vs. NA: 144 vs. 1362 (risk ratio 0.93, 95% CI 0.78 to 1.11)	Mortality risk did not differ significantly between GA and NA.

*GA* general anaesthesia, *NA* neuraxial anaesthesia, *RA* regional anaesthesia

#### Length of hospital stay

**Fig. 4:**
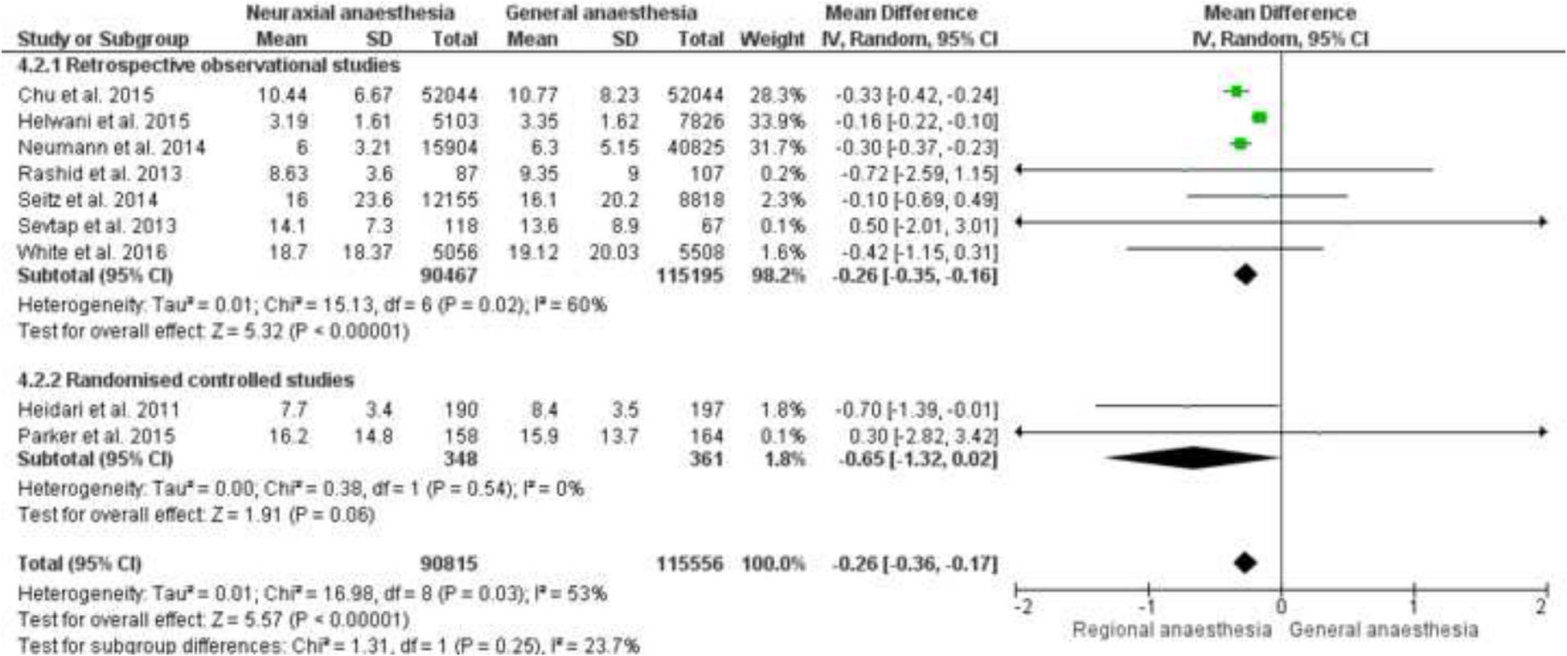
Meta-analysis of the length of hospital stay for the neuraxial anaesthesia group versus the general anaesthesia group calculated with a random effect method. 4.2.1: Mean difference of the retrospective observational studies represented as a subgroup. 4.2.2: Mean difference of the randomised controlled studies represented as a subgroup

Twelve studies examined the length of hospital stay (LOS), see Table 6. [8, 10, 14, 15, 17–20, 25, 26, 31, 33]. Six studies revealed no difference in length of hospital stay related to the anaesthetic technique [14, 17, 19, 20, 26, 31]. One study reported that general anaesthesia was associated with a shorter length of stay [Hazard Ratio (HR): 1.28, 95% CI (1.22 to 1.34); *p* < 0.001] [8]. In contrast five other studies showed that neuraxial anaesthesia was associated with a shorter length of hospital stay [10, 15, 18, 25, 33]. Nine of the twelve studies were included in the meta-analysis [10, 15, 17, 19, 20, 25, 26, 31, 33]. In the meta-analysis we found a significantly shorter length of stay in the neuraxial anaesthesia group [Mean Difference (MD): -0.26; 95% CI (−0.36 to −0.17); *p* < 0,00001]. The heterogeneity was moderate with I^2^ = 53%, see Fig. 4. We made a separate subgroup analysis for the retrospective observational and the randomised controlled studies. The subgroup analysis for the retrospective observational studies indicates a significant shorter length of stay in the hospital in the neuraxial anaesthesia group. [MD -0.26, 95% CI (−0.35 to −0.16), *p* < 0.00001]. The subgroup analysis for the randomised controlled studies revealed no difference between the two groups. [MD: -0.65, 95% CI (−1.32 to −0.01), *p* = 0.06], see Fig. 4.

**Table 6:** Results of the length of hospital stay

Author/Reference	Study type	Anaesthesia	Sample size	Outcome parameter	Results	Conclusion
Basques et al. 2015 [8]	Retrospective observational study	GA vs. SA	*n* = 9.842 GA = 7.253 (73.7%) SA = 2.589 (26.3%)	Length of hospital stay (LOS)	LOS: GA vs SA: (HR: 1.28, 95% CI 1.22 to 1.34, *p* < 0.001)	GA was associated with a shorter LOS.
Chu et al. 2015 [25]	Retrospective observational study	GA vs. NA (spinal/epidural)	*n* = 104.088 GA = 52.044 NA = 52.044	Length of hospital stay	LOS: GA vs. NA: 10.77 (8.23) vs. 10.44 (6.67), *p* < 0.001	The length of hospital stay was significantly shorter in the neuraxial anaesthesia group.
Heidari et al. [33]	Randomised controlled trial	GA vs. NA (spinal/epidural)	*n* = 387 GA = 197 NA = 190	Length of hospital stay	LOS: GA vs. NA: 8.4 (3.5) vs. 7.7 (3.4)	The length of hospital stay was significantly shorter in the NA group.
Helwani et al. 2015 [10]	Retrospective observational study	GA vs NA (SA/EA)	*n* = 12.929 GA = 7.826 (60.5%) NA = 51.03 (39.5%) [SA = 4.377 (85.8%); EA = 126 (2.5%)]	Length of hospital stay (LOS)	LOS: OR = 0.73; 95% CI = 0.68 to 0.89, *p* < 0.001	NA anaesthesia was associated with a reduction in LOS.
Le-Wendling et al. 2012 [14]	Retrospective observational study	GA vs. RA (single injection spinal, continuous spinal, continuous epidural) with or without continuous nerve block	*n* = 308 GA = 235 (76.30%); RA = 73 (23.70%)	Length of hospital stay (LOS)	LOS: RA vs. GA: 6.4 vs. 6.6 days, *p* = 0.64	There was no difference in the length of hospital stay.
Neuman et al. 2014 [15]	Retrospective observational study	GA vs. NA (spinal/epidural)	*n* = 56.729 GA = 40.825 (72%) NA = 15.904 (28%)	Length of hospital stay (LOS)	LOS: NA = 6 days (95% CI: 6 to 6.1) vs. GA = 6.3 days (95% CI: 6.2 to 6.3), *p* < 0.001	NA was associated with modestly shorter LOS.
Parker et al. 2016 [31]	Randomised controlled study	GA vs. SA	*n* = 322 GA = 164 (50.93%) SA = 158 (49.07%)	Length of hospital stay (LOS)	LOS in days (standard deviation): GA = 15.9 (13.7); SA = 16.2 (14.6); *p* = 0.75	There was no difference in the length of hospital stay.
Rashid et al. 2013 [17]	Retrospective observational study	GA vs. NA(epidural/spinal)	*n* = 194 GA = 107 (55.15%) NA = 87 (44.85%)	Length of hospital stay (LOS)	LOS: GA = 9.35 ± 9.0, NA = 8.63 ± 3.6, *p* = 0.484	There were no statistic differences between LOS.
Shih et al. 2010 [18]	Retrospective observational study	GA vs. SA	*n* = 335 GA = 167 (49.85%) SA = 168 (50.15%)	Length of hospital stay (LOS)	LOS:GA vs. SA 9 (4-45) days vs. 8 (2-92) days, *p* = 0.04	The LOS was significantly shorter in the spinal anaesthesia group.
Seitz et al. 2014 [19]	Retrospective observational study	GA (inhalational, intravenous, GA combined with epidural or local anaesthesia) vs. SA	*n* = 20.973 GA = 8.818 (42.1%) SA = 12.155 (57.9%)	Length of hospital stay (LOS)	LOS in days (± standard deviation): GA = 16.1 (20.2), SA = 16.0 (23.6), *p* = 0.72	There was no difference in the length of hospital stay
Sevtap et al. 2013 [20]	Retrospective observational study	GA vs. SA vs. EA	*n* = 185 GA = 67 (36.21%) SA = 67 (36.21%) EA = 51 (27.58%)	Length of hospital stay (LOS)	LOS: GA = 13.6 ± 8.9, SA = 12.5 ± 5.2, EA = 15.7 ± 9.4, *p* = 0.228	There was no difference in the length of hospital stay
White et al. 2016 [26]	Retrospective observational study	GA vs. SA (with or without peripheral nerve block)	*n* = 10,564 GA = 5508 SA = 5056	Length of hospital stay (SD)	LOS: GA vs. SA: 19.12 (20.03) vs. 18.70 vs. 18.37	There was no difference in the length of hospital stay

*EA* epidural anaesthesia, *GA* general anaesthesia, *LOS* length of hospital stay, *NA* neuraxial group, *RA* regional anaesthesia, *SA* spinal anaesthesia, *SD* standard deviation

### Secondary outcomes

#### Cardiac complications/myocardial infarction

Three studies examined the total rate of any cardiac complications after hip fracture surgery [10, 13, 27]. Helwani and colleagues reported that neuraxial anaesthesia is associated with a decreased risk of postoperative cardiac [OR: 0.61; 95% CI (0.44 to 0.85); *p* < 0.001] complications [10]. Kim and colleagues and Neuman and colleagues determined that the methods of anaesthesia did not influence the rate of cardiac complications [13, 27].

**Fig. 5:**
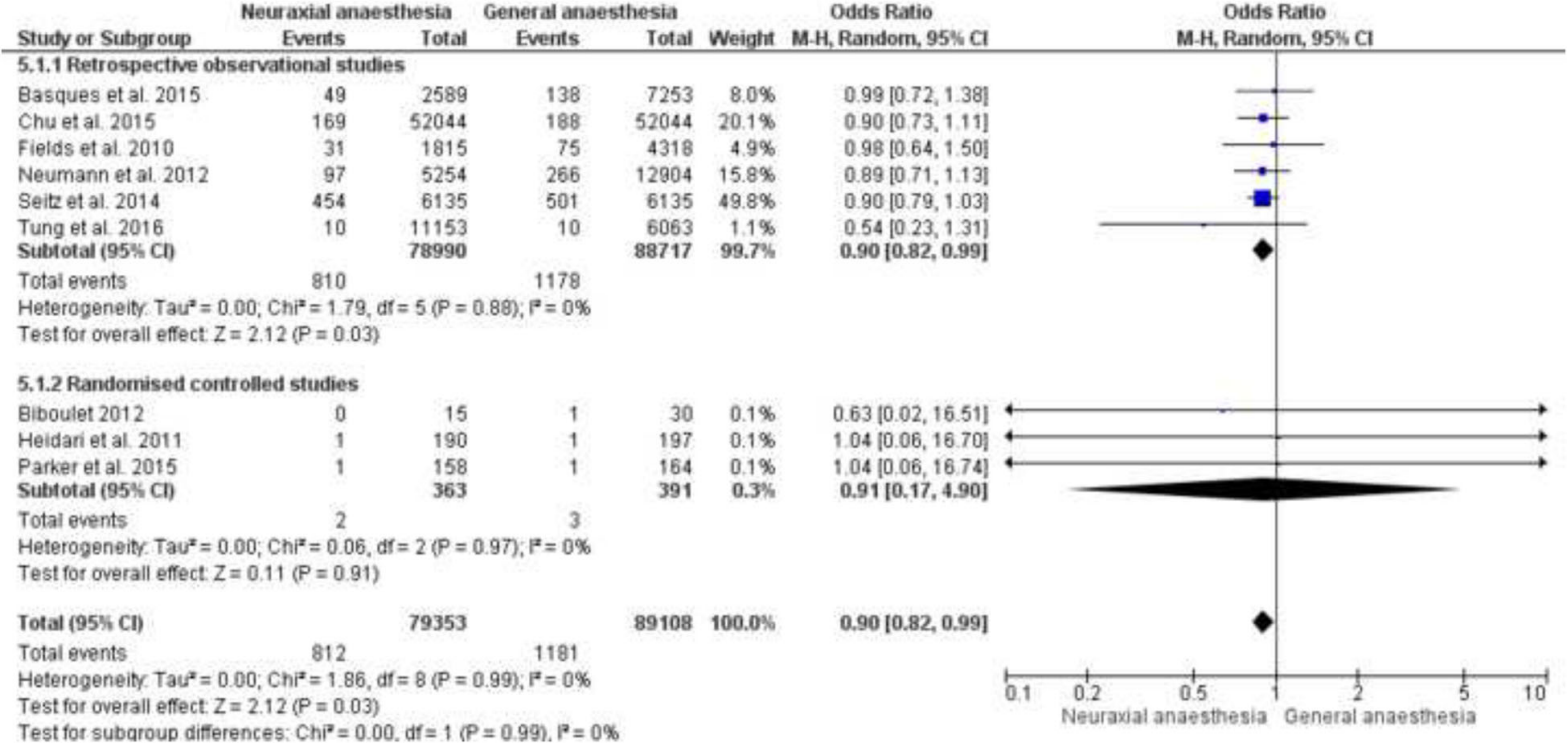
Meta-analysis of myocardial infarction in the neuraxial anaesthesia group versus general anaesthesia group calculated with a random effect method. 5.1.1: Odds ratio of the retrospective observational studies represented as a subgroup. 5.1.2: Odds ratio of the randomised controlled studies represented as a subgroup analysis

Ten studies examined the incidence of myocardial infarction after hip fracture surgery in the general anaesthesia and the neuraxial anaesthesia groups, see Table 7 [8, 9, 19, 22, 24, 25, 27, 31–33]. All studies are in unison that there is no difference between the two groups. Nine studies could be included in a meta-analysis [8, 9, 19, 24, 25, 31–33]. The meta-analysis came to the result that the incidence of postoperative myocardial infarction is significant higher in the general anaesthesia group [OR 0.90, 95% CI (0.82 to 0.99), *p* = 0.03], see Fig. 5. The separate subgroup analysis for the retrospective observational studies revealed a significant higher incidence of myocardial infarction in the general anaesthesia group [OR 0.90, 95% CI (0.82 to 0.99), *p* = 0.03]. The subgroup of the three randomised controlled studies represents no difference between the two groups [OR 0.91, 95% CI (0.17 to 4.90), *p* = 0.91]. The heterogeneity was in all cases I^2^ = 0.

**Table 7:** Results of myocardial infarction

Author/Reference	Study type	Anaesthesia	Sample size	Outcome parameter	Results	Conclusion
Basques et al. 2015 [8]	Retrospective observational study	GA vs. SA	*n* = 9.842 GA = 7.253 (73.7%) SA = 2.589 (26.3%)	Myocardial infarction	SA vs. GA: 1.9% vs. 1.9%; OR 1.00, 95% CI 0.71 to 1.39, *p* = 0.510	The incidence of myocardial infarction was similar in the two groups.
Biboulet et al. 2012 [32]	Randomised controlled study	GA vs. SA (propofol, sevoflurane)	*n* = 45 GA = 30 SA = 15	Myocardial infarction	SA vs. GA: 0 vs. 1, *p* = 1.0	The incidence of myocardial infarction was similar between the two groups.
Chu et al. 2015 [25]	Retrospective observational study	GA vs. NA (spinal/epidural)	*n* = 104.088 GA = 52.044 NA = 52.044	Myocardial infarction	NA vs. GA: 169 (0.32%) vs. 188 (0.36%), *p* = 0.31	The incidence of myocardial infarction was similar between the two groups.
Fields et al. 2010 [9]	Retrospective observational study	GA vs. SA	*n* = 6.133 GA = 4.318 (72.6%) SA = 1.815 (27.4%)	Myocardial infarction	SA vs. NA: 1.71% vs. 1.75%, *p* = 0.92	The incidence of myocardial infarction was similar between the two groups.
Heidari et al. 2011 [33]	Randomised controlled study	GA vs. NA (EA/SA)	*n* = 270 GA = 197 NA = 190	Myocardial infarction	NA vs. GA: 1 (0.6%) vs. 1 (0.5%),	The incidence of myocardial infarction was similar between the two groups.
Neuman et al. 2012 [27]	Retrospective observational study	GA vs. NA	*n* = 18.158 GA = 12.904 NA = 5.254	Myocardial infarction	NA vs. GA: 97 (1.9%) vs. 266 (2.1%), *p* = 0.348	The incidence of myocardial infarction was similar between the two groups.
Parker et al. 2015 [31]	Randomised controlled study	GA vs. SA	*n* = 322 GA = 164 (50.93%) SA = 158 (49.07%)	Myocardial infarction	SA vs. GA: 1 (0.6%) vs. 1 (0.6%), *p* = 1.0	The incidence of myocardial infarction was similar between the two groups.
Seitz et al. 2014 [19]	Retrospective observational study	GA (inhalational, intravenous, GA combined with epidural or local anaesthesia) vs. SA	*n* = 20.973 GA = 8.818 (42.1%) SA = 12.155 (57.9%)	Myocardial infarction	SA vs. GA: 454 (7.4%) vs. 501 (8.2%), *p* = 0.07	The incidence of myocardial infarction was similar between the two groups.
Tung et al. 2016 [24]	Retrospective observational study	GA vs. RA (epidural/spinal)	*n* = 17.189 GA = 6.063 (35.1%) NA = 11.153 (64.9%)	Myocardial infarction	NA vs. GA: 10 (0.1%) vs. 10 (0.1%), *p* = 0.162	The incidence of myocardial infarction was similar between the two groups.
Whiting et al. 2015 [22]	Retrospective observational study	GA vs. SA	*n* = 7.764 GA = 5.840 SA = 1.813	Myocardial infarction	SA vs. GA: Odds ratio 0.84; 95% CI 0.50-1.43, *p* = 0.532	The incidence of myocardial infarction was similar between the two groups.

*CI* confidence interval, *GA* general anaesthesia, *NA* neuraxial anaesthesia, *OR* odds ratio, *SA* spinal anaesthesia, *RA* regional anaesthesia

#### Pulmonary complications

Three studies examined the total rate of pulmonary complications after hip fracture surgery [10, 13, 27]. Helwani and colleagues and Neuman and colleagues reported that neuraxial anaesthesia is associated with a decreased risk of postoperative pulmonary complications [OR 0.51; 95% CI (0.33 to 0.81); *p* < 0.01] [OR 0.752, 95% CI (0.637 to 0.887); *p* < 0.0001] [10, 27]. Kim and colleagues determined that the methods of anaesthesia did not influence the rate of pulmonary complications [13].

**Fig. 6:**
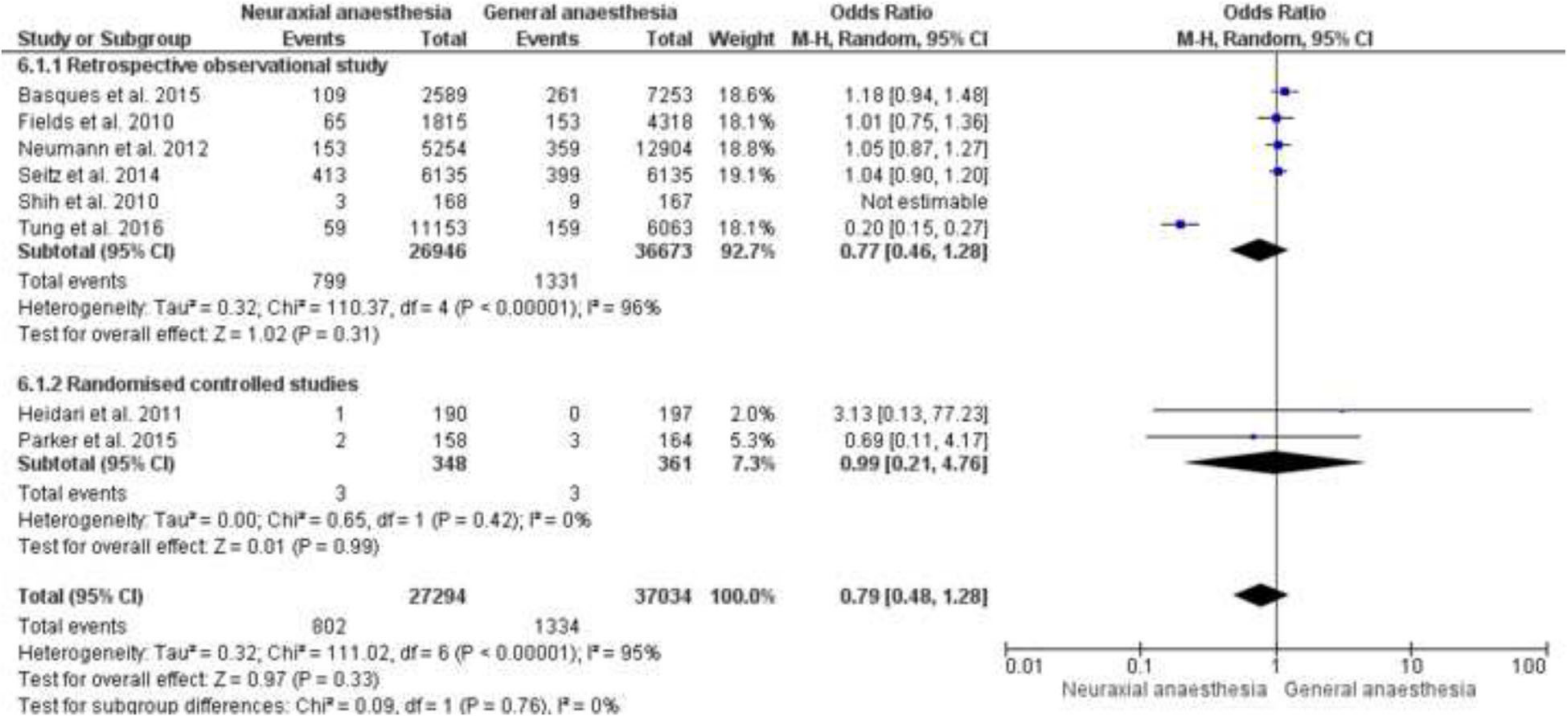
Meta-analysis of pneumonia in the neuraxial anaesthesia group versus the general anaesthesia group calculated with a random effect method. 6.1.1: Odds ratio of the retrospective observational studies represented as a subgroup. 6.1.2: Odds ratio of the randomised controlled studies represented as a subgroup

Nine studies analysed the incidence of pneumonia after general and neuraxial anaesthesia in patients with a hip fracture, see Table 8 [8, 9, 18, 19, 22, 24, 27, 31, 33]. The study of Shih and colleagues and Tung and colleagues revealed a significant higher incidence of pneumonia in the general anaesthesia group [18, 24]. The other studies showed no difference between the two groups [8, 9, 19, 22, 27, 31, 33]. Eight of the nine studies could be included in a meta-analysis, see Fig. 6 [8, 9, 18, 19, 22, 24, 31, 33]. The meta-analysis revealed a similar incidence of pneumonia between the general and the neuraxial anaesthesia groups [OR 0.74, 95% CI (0.46 to 1.17), *p* = 0.20]. The heterogeneity is high (I^2^ = 94%). The separate subgroup analysis of the retrospective observational studies revealed no significant difference between the examined groups [OR 0.72, 95% CI (0.44 to 1.17), *p* = 0.18]. The subgroup analysis of the randomised controlled indicate no difference between the two groups [OR 0.99, 95% CI (0.21 to 4.76), *p* = 0.99].

**Table 8:** Results of the incidence of pneumonia, pulmonary embolism and respiratory

Author/Reference	Study type	Anaesthesia	Sample size	Outcome parameter	Results	Conclusion
Basques et al. 2015 [8]	Retrospective observational study	GA vs. SA	*n* = 9.842 GA = 7.253 (73.7%) SA = 2.589 (26.3%)	Pneumonia	SA vs. GA: 4.2% vs. 3.6%, OR 0.84, 95% CI 0.67 to 0.1.07, *p* = 0.154	The incidence of pneumonia was similar between the two groups.
Chu et al. 2015 [25]	Retrospective observational study	GA vs. NA (spinal/epidural)	*n* = 104.088 A = 52.044 NA = 52.044	Acute respiratory failure	NA vs. GA: 328 (0.63%) vs. 868 (1.67), *p* < 0001	The incidence of respiratory failure was significantly lower in the neuraxial group.
Fields et al. 2010 [9]	Retrospective observational study	GA vs. SA	*n* = 6.133 GA = 4.318 (72.6%) SA = 1.815 (27.4%)	Pneumonia Pulmonary embolism	Pneumonia: SA vs. GA: 3.58% vs. 3.55%, *p* = 0.96; Pulmonary embolism: 0.45% vs. 0.89%, *p* = 0.10	The incidence of pneumonia and pulmonary embolism was similar between the two groups.
Heidari et al. 2011 [33]	Randomised controlled study	GA vs. NA (EA/SA)	*n* = 270 GA = 197 NA = 190	Pneumonia	NA vs. GA: 1 (0.6%) vs. 0	The incidence of pneumonia was similar between the two groups.
Neuman et al. 2012 [27]	Retrospective observational study	GA vs. NA	*n* = 18.158 GA = 12.904 NA = 5.254	Pneumonia, Respiratory failure	Pneumonia: NA vs. GA: 153 (2.9%) vs. 359 (2.8%), *p* = 0.631;Respiratory failure: 180 (3.4%) vs. 641 (5.0%), *p* < 0.0001	The incidence of pneumonia was similar in both groups. The incidence of respiratory failure was significant lower in neuraxial anaesthesia group.
Parker et al. 2015 [31]	Randomised controlled study	GA vs. SA	*n* = 322 GA = 164 (50.93%) SA = 158 (49.07%)	Pneumonia Pulmonary embolism	Pneumonia: SA vs. GA: 2 (1.3%) vs. 3 (1.8%), *p* = 1.0; Pulmonary embolism: 0 vs. 2 (1.2%), *p* = 0.50	The incidence of pneumonia and pulmonary embolism was similar in both groups.
Seitz et al. 2014 [19]	Retrospective observational study	GA (inhalational, intravenous, GA combined with epidural or local anaesthesia) vs. SA	*n* = 20.973 GA = 8.818 (42.1%) SA = 12.155 (57.9%)	Pneumonia Pulmonary embolism	Pneumonia: SA vs. GA: 413 (6.7%) vs. 399 (6.5%), *p* = 0.61; Pulmonary embolism: 49 (0.9%) vs. 67 (1.1%) *p* = 0.09	The incidence of pneumonia and pulmonary embolism was similar in both groups.
Shih et al. 2010 [18]	Retrospective observational study	GA vs. SA	*n* = 335 GA = 167 (49.85%) SA = 168 (50.15%)	Pneumonia, Respiratory failure	Pneumonia: SA vs. GA: 3 vs. 9; Respiratory failure: 0 vs. 1	The incidence of pneumonia was significantly higher in the general anaesthesia group. The incidence of respiratory failure was similar between the two groups.
Tung et al. 2016 [24]	Retrospective observational study	GA vs. RA (epidural/spinal)	*n* = 17.189 GA = 6.063 (35.1%) NA = 11.153 (64.9%)	Pneumonia	NA vs. GA: 59 (1.0%) vs. 159 (1.4%), *p* = 0.012	The incidence of pneumonia was significantly higher in the general anaesthesia group.
Whiting et al. 2015 [22]	Retrospective observational study	GA vs. SA	*n* = 7.764 GA = 5.840 SA = 1.813	Pneumonia Pulmonary embolism	Pneumonia: SA vs. GA: Odds ratio 1.19, 95% CI 0.83 to 1.71, *p* = 0.337; Pulmonary embolism: OR 0.48, 95% CI 0.18 to 1.23, *p* = 0.129	The incidence of pneumonia and pulmonary embolism was similar between the two groups.

*CI* confidence interval, *GA* general anaesthesia, *NA* neuraxial anaesthesia, *OR* odds ratio, *SA* spinal anaesthesia, *RA* regional anaesthesia

**Fig. 7:**
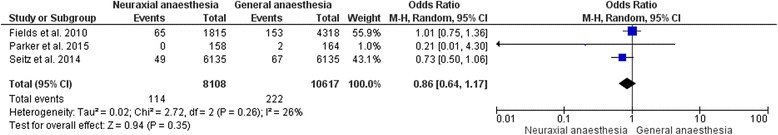
Meta-analysis of pulmonary embolism in the neuraxial anaesthesia group versus the general anaesthesia group. The odds ratio was calculated with a random effect method

The incidence of postoperative pulmonary embolism was examined by four studies, see Table 8 [9, 19, 22, 31]. All studies revealed no difference between the general and the neuraxial groups. The study of Fields and colleagues, Parker and colleagues and Seitz and colleagues could be included in a meta-analysis, see Fig. 7 [9, 19, 31]. The meta-analysis showed no significant difference in the incidence of pulmonary embolism between the two groups [OR 0.86, 95% CI (0.64 to 1.17), *p* = 0.35].

**Fig. 8:**
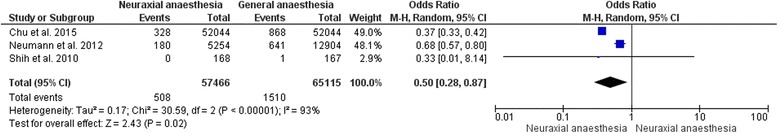
Meta-analysis of the respiratory failure in the neuraxial anaesthesia group versus the general anaesthesia group. The odds ratio was calculated with a random effect method

The incidence of respiratory failure was tested by three studies, see Table 8 [18, 25, 27]. The studies of Chu and colleagues and Neuman and colleagues indicated a significant higher incidence of respiratory failure in the general anaesthesia group [25, 27]. The study of Shih and colleagues revealed no difference between the two groups. All three studies were included in a meta-analysis [18, 25, 27]. The meta-analysis showed a significant lower incidence of respiratory failure in the neuraxial anaesthesia group [OR 0.50, 95% CI (0.28 to 0.87), *p* = 0.02], see Fig. 8.

## Discussion

In our systematic review and meta-analysis we included 23 studies with 413.999 patients. 249.408 patients received general anaesthesia and 150.964 neuraxial anaesthesia (epidural/spinal). We could not detect any difference in the 30-day mortality in patients undergoing hip fracture surgery. However, the length of hospital stay and the in-hospital mortality were significantly shorter in the neuraxial anaesthesia group. Of the secondary outcomes the incidence of myocardial infarction and respiratory failure was significant lower in the neuraxial anaesthesia group. There was no difference in the incidence of pneumonia between the two groups. Of note, out of the 23 studies which met our inclusion criteria, 20 were mainly large retrospective observational studies and three were prospective randomised [8–30]. In 2010 a systematic review carried out by Luger and colleagues examined the type of anaesthesia in hip fracture surgery. They included literature from the years 1967 to 2010 in their systematic review. They were able to include 34 randomised studies, 14 observational studies and 8 systematic reviews and meta-analyses in their study. The authors speculated that spinal anaesthesia may be associated with significantly reduced early mortality, fewer incidents of deep vein thrombosis, less acute postoperative confusion, a tendency to fewer myocardial infarction, fewer cases of pneumonia, fatal pulmonary embolism and postoperative hypoxia [34]. However, the review was limited, as only 18.715 patients were included. With regard to the limited evidence the authors concluded that, neither general, nor regional anaesthesia seem to improve perioperative outcome [34]. To the best of our knowledge, the most recent effort to bundle information in a systematic review addressing the type of anaesthesia in hip fracture surgery has been performed in 2016 by Guay and colleagues in a Cochrane Review. They included only randomised studies from 2003 to 2014. In total 31 studies were included with 3231 patients. Furthermore, only 2152 patients were available to examine the 30-day mortality. They did not find a difference between the two techniques. The authors determined that the number of patients included in the study was insufficient to reveal a difference between general and regional anaesthesia in hip fracture patients [7]. For these reasons we decided to include both prospective randomised and retrospective observational studies to assess as many patients as possible for the systematic review and the meta-analyses. In our systematic review and meta-analysis fourteen studies assessed the 30-day mortality. However, the high number of patients is limited through the high risk of selection bias.

As mentioned above two studies assessed the overall-mortality. Karaman and colleagues revealed that there is a higher mortality rate after receiving general than neuraxial anaesthesia [12]. Shih and colleagues concluded that there is no significant difference between the general and the neuraxial anaesthesia group [18]. Though, there are some limitations. For Karaman and colleagues the overall-mortality was defined as the mortality rate during the length of stay and the follow-up time [12]. Follow-up time was defined as the time period of the study duration. The follow-up time fluctuated between zero and 60 months [12]. For Shih and colleagues overall-mortality means the incidence of death since discharge. Obviously it is not possible to compare these two overall-mortalities. The neuraxial anaesthesia group of Karaman and colleagues included spinal and epidural anaesthesia [12]. The neuraxial anaesthesia group of Shih and colleagues included only spinal anaesthesia. [19].

Five other studies examined the in-hospital mortality [14, 16, 25, 27, 33]. The study of Chu et al. revealed a significant higher incidence of in-hospital mortality in the general anaesthesia group [25]. The meta-analysis of the in-hospital mortality showed therefore a significant higher incidence of in-hospital mortality in the general anaesthesia group. [14, 16, 25, 27, 33] The study of Chu et al. included 104.088 patients in their study and is weighted in the analysis with 53.2% [25]. The other studies are considerable smaller [14, 16, 27, 33]. The conclusion of the meta-analysis is therefore limited. However the 30-day mortality rate revealed no difference between the groups. It seems like, if the patient survives the hospital stay, the risk to die in the next weeks is equal whatever anaesthesia technique was applied. Due to aforementioned limitations of the meta-analyses, there is an urge of randomised controlled studies examining the effect of anaesthesia technique regarding the in-hospital and 30-day mortality rates, taking into account possible influencing variables like the age of the patients, pre-existing conditions and type of surgery.

We found a significant shorter length of stay in the neuraxial anaesthesia group compared to the general anaesthesia group. We could include nine studies out of twelve [10, 15, 17, 19, 20, 25, 26, 31, 33]. The other three studies did not mentioned the standard deviation [8, 14, 19]. Due to non-response it was impossible to receive the missing parameters from the authors. Only four studies in the meta-analysis revealed a significant shorter length of stay in the neuraxial anaesthesia group. Three of them are the studies getting the most weight in the analysis [10, 15, 25]. Therefore our results have to be interpreted with caution. The meta-analysis revealed that the length of hospital stay is one quarter of a day shorter in the neuraxial anaesthesia group, which most likely has no clinical relevance. Another problem is the different definition of the meaning of length of hospital stay. Most of the authors documented the overall length of stay. In contrast the study of Heidari and colleagues represent the length of stay before and after the surgery [33]. This is an important point, because a delay of surgery extends the length of stay. Helwani and colleagues and Neuman and colleagues showed that neuraxial anaesthesia is associated with a modestly shorter length of stay [10, 15]. In the study of Neuman and colleagues the authors considered the fracture type and the performed surgery procedure [15]. Minimally invasive approaches may decrease the length of stay. Kazemian and colleagues published in 2013 a randomised controlled study examining the treatment of intertrochanteric fractures in elderly patients by a dynamic hip screw and external fixation. The treatment of the hip fracture with the minimal-invasive external fixation showed minimal blood loss, pain reduction, shorter length of hospital stay and favourable functional outcomes compared to the dynamic hip screw [35]. Basques and colleagues revealed that general anaesthesia is associated with a shorter length of stay. However, patients receiving a general anaesthesia were younger, had higher Body Mass Index (BMI) and less comorbidities [8]. The authors used a propensity score to reduce the selection bias and the differences between the two groups, but the length of hospital length of stay was shorter in the general anaesthesia group. The question remains unanswered, if the length of stay in this group was shorter because the patients received general anaesthesia or due to the fact that patients were younger and did not have as many chronic diseases as the older patients who received a spinal anaesthesia. After all the risk of selection bias is high. Another important limitation is the different health care systems of the four included studies for the meta-analysis. The study of Seitz and colleagues and Helwani and colleagues were performed in the United States of America, the study of Sevtap and colleagues in Turkey and the study of Rashid and colleagues in Pakistan [10, 17–20]. Caution is advised when comparing the length of hospital stay of patients with hip fracture in different countries with differing health care systems and discharge points.

In addition we investigated the incidence of myocardial infarction, pneumonia and pulmonary embolism between general and neuraxial anaesthesia after hip fracture surgery. The meta-analysis of the myocardial infarction revealed a significant higher incidence of myocardial infarction in the general anaesthesia group. However, the meta-analysis has got several limitations. All nine studies showed no difference between the two groups. A considerable bias was introduced by the retrospective observational studies. The result of the meta-analysis has to be interpreted with caution.

The meta-analysis of the incidence of postoperative pneumonia indicated no difference between the general and neuraxial anaesthesia groups. Only the study of Shih and colleagues revealed a significant higher incidence of pneumonia in the general anaesthesia group. However the study had only a small sample size of 335 patients [18]. Kamel and colleagues examined 2003 in a study the time to ambulation (walking) after hip fracture surgery. In this study the type of anaesthesia had no influence of the time to ambulation after a hip fracture surgery. However, a prolonged time to ambulation was associated with a longer length of stay and a higher incidence of pneumonia [36].

The meta-analysis of the incidence of the pulmonary embolism showed no significant difference between the general and the neuraxial anaesthesia group. The validity of the meta-analysis regarding pulmonary embolism is limited by the inclusion of only four studies.

This systematic review and meta-analysis has several important limitations. 20 from 23 included studies were retrospective observational studies and only three were randomised [8–27, 31–33]. Retrospective studies have a high risk for selection bias, confounding factors and unobserved differences between the neuraxial and the general anaesthesia group. Most of the authors used a propensity score to reduce the risk of bias, but randomised studies would be preferable [8–16, 18–26]. On the other hand the retrospective studies in this review included overall 413.999 patients. The data for the studies were obtained from databases like ACS-NSQIP (American College of Surgeons National Quality Improvement program). The database considered 135 variables, including preoperative risk factors, 30-day mortality, overall-mortality, surgical site infection etc. [37]. Many patients had to be excluded because of incomplete documents. Most of the studies did not describe the dosage and the type of the anaesthetic used. Only the paper of Shih and colleagues and Sevtap and colleagues described the dosage and the type of anaesthetic in detail [18, 20]. In 20 of the 23 studies no information is available whether patients with neuraxial anaesthesia received additional sedation. Shih and colleagues, White and colleagues and Heidari and colleagues are the only authors who mentioned if there was a sedation used [18, 26, 33]. The sample size of the included studies varied widely. However, RevMan weights the studies according to the precision of the effect size. Therefore, we decided to include also small studies. Another limitation is the restricted set of outcomes. We were not able to examine functional outcome or disability-free survival. In addition, it would be advisable for future systematic reviews and meta-analyses to take studies assessing the effect of types of surgery [38], applied anaesthetics and their dosage [39] into consideration.

## Conclusion

In this meta-analysis we could not observe any difference in the 30-day mortality rate between neuraxial and general anaesthesia. Length of hospital stay and the in-hospital mortality was shorter in the neuraxial anaesthesia group. There is an urgent need to carry out large randomised studies, which will reflect “real world” approaches to general and neuraxial anaesthesia, like e.g. the REGAIN trial (www.regaintrial.org.).

## Abbreviations

ACS-NSQIP: American College of Surgeons National Quality Improvement program
AS: Ana Stevanovic
BMI: Body mass index
CI: Confidence Interval
Cvc: Cardiovascular complications
Dssi: Deep surgical site infection
EA: Epidural anaesthesia
GA: General anaesthesia
HR: Hazard ratio
Hz: Hospitalization costs
I^2^: Heterogeneity
ICTRP: International clinical trials registry platform
IQR: Interquartile range
JVW: Julia Van Waesberghe
LOS: Length of hospital stay
MC: Mark Coburn
MD: Mean difference
NA: Neuraxial anaesthesia
OR: Odds ratio
Pc: Pulmonary complications
RA: Regional anaesthesia
RR: Rolf Rossaint
SA: Spinal anaesthesia
SD: Standard deviation
